# Medical Assistance in Dying in Psychiatry, An Ethical Analysis.

**DOI:** 10.1192/j.eurpsy.2023.402

**Published:** 2023-07-19

**Authors:** K. Keenan

**Affiliations:** Department of Psychiatry, Connolly Hospital, Dublin, Ireland

## Abstract

**Introduction:**

Assisted dying (AD) is a general term in the literature to incorporate both physician-assisted suicide (PAS) and voluntary active euthanasia. In 2002 Belgium became the first country in the world to specifically acknowledge mental suffering in law as a valid basis for AD, specifically euthanasia, with other countries passing similar AD legislation more recently. Local legislation stipulates both substantive and procedural criteria that must be met for AD in jurisdictions, with only minor differences in procedural criteria noted accross sites.

In countries without AD legislation it remains a criminal offence for a physician to partake in AD, the offence prosecutable under local laws as manslaughter. It is a fiercely contentious issue within the medical, legal, political, religious and ethical fields with lack of consensus and on-going deliberation.

**Objectives:**

The author examines literature regarding the ethical issues raised by medical assistance in dying in psychiatry.

**Methods:**

A non-systematized review of the literature, using literature available on PubMed, PsychINFO and Medline.

**Results:**

Findings from this review indicate that Beauchamp & Childress’ biomedical approach of equilibrating the ethical principles of ‘respect for autonomy’, ‘beneficience’, ‘nonmaleficence’ and ‘justice’, to act in the best interests of their patients are those most used in contemporary psychiatric practice. There is a fundamental theme suggested in the literature that ‘respect for autonomy’ is both the prevailing and challenging ethical principle to soundly navigate in AD cases. Within this principle, the task of objectively assessing capacity remains dominant.

Psychiatry remains unique in its pathology, biological and social entanglement hence the literature suggests a limit to autonomous decisions be considered, due to the extreme vulnerability and vast potential for abuse of this patient cohort.

Ultimately, the literature suggests physicians adhere to available professional medical ethical guidelines (should they be available), using an objective scale for undertaking capacity assessments, and seeking advice from the courts rather than bearing any outstanding ethical burden in these most complex of cases.

**Conclusions:**

Infinite complexities and dissensus surrounding practice of psychiatric AD. The ethical principle of autonomy retains a significant role in both AD and psychiatric debates, with specific attention drawn the quandary of psychiatric capacity assessment. In addition to the moral question of whether it is appropriate to assist psychiatric patients to end their lives, the appropriateness of this role for psychiatrists is yet to be determined in light of professional disdain. Further analysis of cases are required, as they are published over time, to further reform the ethical and legal arguments.

**Disclosure of Interest:**

None Declared

